# The business of dietetics: Results from the national Australian private practice dietetics dataset

**DOI:** 10.1111/1747-0080.70036

**Published:** 2025-08-07

**Authors:** Amy Kirkegaard, Peter Clark, Olivia R. L. Wright, Lauren Ball

**Affiliations:** ^1^ Centre for Community Health and Wellbeing The University of Queensland Springfield Queensland Australia; ^2^ School of Human Movement and Nutrition Sciences The University of Queensland Springfield Queensland Australia; ^3^ Faculty of Health Sciences and Medicine Bond University Queensland Australia; ^4^ School of Public Health The University of Queensland St Lucia Queensland Australia

**Keywords:** cross‐sectional studies, dietetics, health services administration, private practice, workforce

## Abstract

**Aims:**

This study aimed to describe private dietetics practices in Australia in terms of ownership, size, and operation, team composition, and products and services.

**Methods:**

Australian dietitians operating private practices were invited to participate in a cross‐sectional survey between May and June 2024. The survey captured data regarding the business, including ownership, revenue, business and clinical operation, team composition and remuneration, products and services, and consultation and program characteristics. Data were analyzed descriptively.

**Results:**

One hundred and fifty‐four participants completed the survey (22% response rate). Businesses were primarily sole traders (*n* = 105; 68%) that neither employed nor contracted health professionals (*n* = 94; 61%) and generated less than AU$149999 in revenue (*n* = 114; 77%). Most businesses specialised (*n* = 107; 69%), with common areas of specialisation being eating disorders (*n* = 49; 32%), disability support (*n* = 35; 23%), and weight inclusive care (*n* = 35; 23%). Client consultations (*n* = 146; 95%) were the most frequently offered service. Initial consultations were most frequently 60 min (*n* = 83; 59%), attracting a mean fee of AU$173 (±$9 95% CI), while review consultations were most frequently 30 min (*n* = 80, 57%) and AU$113 (±$7 95% CI). Business and dietitian performance evaluation were typically not performed periodically, yet both were positively correlated with revenue (*τ*_b_ = 0.231, *p* < 0.001 and *τ*_b_ = 0.338, *p* < 0.001, respectively).

**Conclusions:**

Sole practitioners represent a significant proportion of private dietetics practice. With a significant proportion of the workforce working in this area, support is needed to ensure private practices are sustainable and the workforce can continue to provide much‐needed services for Australians.

## 1 INTRODUCTION

Private practice is the largest area of practice for dietitians in Australia, with estimates suggesting between a third to half of new and experienced practitioners are active in this area.^1^, ^2^, ^3^ Yet, knowledge on private practice dietetics has not kept pace with its rapid expansion, leading to topics within private practice that are well understood, such as dietitian and client experiences and preferences,^4^ and others where our understanding is lacking. A current and comprehensive understanding of the business of private practice dietetics is essential to inform workforce planning and advocacy efforts.^5^, ^6^

Topics within private practice that have drawn considerable attention have primarily relied on consumers' and stakeholders' perspectives to explore experiences and preferences in care delivery.^7^, ^8^, ^9^, ^10^, ^11^ Research in this area has focused on the delivery of high quality,^8^ patient‐centred care,^9^, ^10^ with a focus on accessibility in terms of mode, cost, and coordination of care.^7^ Dietitians have also voiced concerns regarding care coordination and government subsidies, such as the Medicare Benefits Schedule.^12^, ^13^, ^14^ Similarly, general practitioners have identified challenges impacting referral to dietitians,^15^ including cost, patient interest or commitment, and knowledge gaps regarding the scope and benefits of dietetic care.^15^, ^16^, ^17^ Despite this body of research, key knowledge gaps remain, including contemporary data pertaining to practice distribution, products and services offered by practices, and the setting, mode, and fees paid for those products and services.

More recently, research has focused on the operational aspects of private practice, including the organisational systems required to support quality and evaluation.^18^, ^19^, ^20^ For example, Kirkegaard et al. developed a system‐based approach to enhancing quality in private practice^18^ that was informed by national practice standards, while Clark et al. identified priority clinical and business standards that practices should collect and showed they are feasible to implement in private practice.^20^, ^21^ However, the maturity of business systems and the degree to which practices routinely evaluate performance are largely unknown. More broadly, dietitians have identified gaps in business acumen, knowledge, and skills, which are essential to operating a high‐quality and sustainable private practice.^4^, ^22^, ^23^ This knowledge is essential to inform the development of resources and tools to support dietitians to operate effective and sustainable practices.

Despite the increasing number of dietitians working in private practice and the growing research focus in this area, knowledge gaps remain, even in topics that have experienced more intense research investment. Little is known about the organisational contexts in which private practice dietitians work, with no studies providing a comprehensive description of private practices as entities. Therefore, this study aimed to describe private dietetics practices in Australia in terms of ownership, revenue, business and clinical operation, team composition and remuneration, and products and services. In doing so, this study will provide contemporary, real‐world data to address key knowledge gaps previously described.

## 2 METHODS

This pragmatic study draws upon baseline, cross‐sectional data from the inaugural survey for a national dataset describing the workforce and businesses comprising private practice dietetics in Australia. For the purposes of the national dataset, a “private practice” was defined as “a professional business that is not controlled or paid for by the government or larger organisation (such as a hospital)”.^24^ Detailed methods and workforce findings are reported elsewhere.^25^ Briefly, the survey was developed by the research team to capture data describing the workforce (e.g., employment status and settings, activities performed, and time investment) and business (e.g., ownership, revenue, business and clinical operation, team composition and remuneration, and products and services). The survey was pretested with seven private practice dietitians and changes made to the survey structure and item wording. Pretest data were not included in the study. The final survey contained 123 questions; however, use of branch logic meant that participants were only presented with questions relevant to them.

Private practices and their owners were compiled into a register using the “Find a Dietitian” search tool administered by Dietitians Australia, with each entry validated using the Google search engine. The final register contained details for 703 private practices. Invitations were emailed to practice owners beginning in May 2024. The initial invitation described the study and invited participants to complete the survey via an electronic link. Additional information about eligibility was sent via a second email approximately 2 weeks later. Reminders were emailed 1 week, 3 days, and 1 day prior to survey closure. A participant information sheet was provided with the initial email, and consent was obtained on the first page of the survey. Participants were offered a summary report for use as a benchmarking tool, to be provided after survey closure.

Data from the business section of the survey were analysed using IBM SPSS Statistics for Windows (version 29.0.2.0; Armonk, NY: IBM Corp).^26^ Fees were calculated as a continuous variable and described using mean and standard deviation to enhance comparison to previous research.^27^ Year business began trading was captured as a continuous variable but was transformed into an ordinal variable for analysis. Categorical variables were described using frequencies and percentages. Associations between nominal and ordinal variables were measured using Pearson's chi‐square test or Kendall's Tau‐b, respectively. For Pearson's chi‐square tests, where possible, variable categories were collapsed so that expected values for at least 80% of cells were five or more. Results identify where this assumption was not met because it was not possible to collapse categories meaningfully. Statistical significance was set at *p* < 0.05. Analysis of short answer responses describing the key challenges experienced in private practice was conducted using content analysis,^28^ with categories related to private practices reported here.

Ethical approval for this study was obtained from the University of Queensland Human Research Ethics Committee (2023/HE001399). This study was reported in accordance with the Strengthening the Reporting of Observational Studies in Epidemiology Statement.^29^

## 3 RESULTS

Seven hundred and three business owners operating a private practice were invited to participate in the census, with 154 participants completing the business section of the survey (22% response rate). Participants who completed the business section were primarily business owners (*n* = 148; 96%), with a small number of employees or contractors (*n* = 6; 4%) completing the survey on behalf of the business owner. Business owners completing the business section were primarily female (*n* = 135; 91%) and aged between 31 and 50 years (*n* = 101; 68%). No significant differences were observed between survey respondents and Dietitians Australia members in terms of gender (*p* = 0.23) or location (*p* = 0.39). A significant difference was observed in terms of age (*p* = <0.001), indicating that the sample was older than Dietitians Australia members.

Characteristics of private practices described by participants in this study are provided in Table 1. Practices were primarily sole traders (*n* = 105; 68%) and generated less than AU$149 999 in revenue in FY2022–2023 (*n* = 114; 77%). The most frequently reported challenge identified in the short‐answer responses related to achieving an adequate and consistent income (*n* = 22), with some participants focusing specifically on the challenge of attracting new clients to the practice (*n* = 21), or existing clients either cancelling or not attending their appointment resulting in lost revenue and income (*n* = 19). Many participants (*n* = 16) identified the cost‐of‐living crisis as being responsible for the observed decline in new clients. Participants (*n* = 16) also noted the negative impact of the cost‐of‐living crisis on profitability due to rising business costs. A majority had been operating for less than 10 years (*n* = 106; 69%), though only a small number (*n* = 6) of participants identified the challenges of establishing the practice, including establishing procedures, marketing, and establishing referral networks, and transitioning from other employment. A significant positive correlation was observed between the year the practice began trading and revenue (*τ*_b_ = 0.346, *p* < 0.001), suggesting revenue increased with business age. A modest but significant association was observed between the complexity of business structure and gross revenue (*χ* ^2^ (4) = 55.87, *p* < 0.001), whereby dietitians in complex business structures (company or trust) were more likely to report higher gross revenue than dietitians with simple business structures (sole trader, partnership). No associations were observed between gross revenue and geographic or regional location (*p* > 0.05), noting that the assumption of expected values was not met for geographic location.

**TABLE 1:** Characteristics of private dietetics practices operating in Australia as described by participants (*n* = 154).

Characteristic	*n* (%)
Business structure	
Sole trader	105 (68)
Company	39 (25)
Trust	7 (5)
Partnership	2 (1)
Prefer not to say	1 (<1)
Number of owners (excluding sole traders)	
1	32 (64)
2	13 (26)
3	2 (4)
4	1 (2)
5	2 (4)
Professions of owners	
Dietitian	46 (66)
Non‐health professional	11 (16)
Exercise physiologist	6 (9)
Physiotherapist	2 (3)
Diabetes educator	1 (1)
Other	4 (6)
Year business began trading	
2024–2020	55 (36)
2019–2015	51 (33)
2014–2010	17 (11)
2009–2005	20 (13)
2004–2000	6 (4)
1999–1995	2 (1)
1994–1990	2 (1)
1989–1985	1 (<1)
Gross revenue in FY2022–2023	
Less than $49 999	46 (31)
$50 000–$99 999	41 (28)
$100 000–$149 999	27 (18)
$150 000–$199 999	4 (3)
$200 000–$299 999	4 (3)
$300 000–$399 999	7 (5)
$400 000–$499 999	0 (0)
$500 000–$599 999	2 (1)
$600 000–$699 999	2 (1)
$700 000–$799 999	6 (4)
$800 000–$899 999	1 (<1)
$900 000–$999 999	1 (<1)
$1 000 000–$1 999 999	4 (3)
$2 000 000–$2 999 999	2 (1)
Geographic location of in‐person services	
New South Wales	42 (27)
Queensland	32 (21)
Victoria	31 (20)
Western Australia	10 (7)
Australian Capital Territory	5 (3)
South Australia	4 (3)
Tasmania	1 (<1)
Multiple states and/or territories	9 (6)
No in‐person services	20 (13)
Regional location of in‐person services	
MM 1 (metropolitan areas)	80 (60)
MM 2 (regional centres)	21 (16)
MM 3 (large rural towns)	4 (3)
MM 4 (medium rural towns)	3 (2)
MM 5 (small rural towns)	2 (2)
Multiple regional locations	24 (18)
Practice specialisation	
Generalised	47 (31)
Eating disorders	49 (32)
Disability support	35 (23)
Weight inclusive care	35 (23)
Food allergies and intolerances	33 (21)
Paediatrics	33 (21)
Restricted/fussy eating	24 (16)
Women's health	23 (15)
Weight management	20 (13)
Diabetes	19 (12)
Reproductive health	19 (12)
Sports nutrition	16 (10)
Malnutrition	15 (10)
Autoimmune disorders	12 (8)
Older adults	12 (8)
Mental health	11 (7)
Gastrointestinal health	10 (6)
Other	13 (8)

Abbreviations: FY, financial year; MM, Modified Monash category.

Practices were generalised in service focus (*n* = 47; 31%), somewhat specialised (*n* = 36; 23%), mostly specialised (*n* = 36; 23%), or specialised (*n* = 35; 23%). Of practices that were specialised to some degree (*n* = 107), common areas of specialisation were eating disorders (*n* = 49; 46%), disability support (*n* = 35; 33%), and weight‐inclusive care (*n* = 35; 33%). Practices that specialised in other areas (*n* = 13; 12%) included neurodivergent, tube‐fed, vegan or vegetarian, renal, and cardiovascular populations or people with hypermobility or invisible illnesses.

Characteristics of the team employed within private practices are presented in Table 2. Most practices neither employed nor contracted other health professionals beyond the business owner (*n* = 94; 61%), though a smaller proportion employed (*n* = 24; 16%), contracted (*n* = 26; 17%), or both employed and contracted (*n* = 10; 7%) other health professionals. All practices that employed health professionals employed dietitians (*n* = 34). However, a small number of practices that contracted health professionals did not contract dietitians (*n* = 3). Dietitians across all experience levels were employed or contracted, with a small number of practices employing or contracting dietitians as clinical managers (*n* = 7; 12%). Remuneration for employed dietitians increased with experience, as illustrated in Table S1. Almost one‐third of practices also employed reception staff (*n* = 47; 31%), with a smaller proportion employing a practice manager (*n* = 13; 8%).

**TABLE 2:** Characteristics of the workforce employed or contracted by private dietetics practices operating in Australia.

Characteristic	Employed	Contracted
Number of health professionals employed (*n* = 34) or contracted (*n* = 36)		
1	9 (26)	11 (31)
2	5 (15)	8 (22)
3	8 (24)	7 (19)
4	1 (3)	6 (17)
5	3 (9)	1 (3)
6	4 (12)	1 (3)
10	2 (6)	0 (0)
12	0 (0)	1 (3)
16	1 (3)	0 (0)
17	0 (0)	1 (3)
28	1 (3)	0 (0)
Health professions employed (*n* = 34) or contracted (*n* = 36)		
Dietitian	34 (100)	33 (92)
Exercise physiologist	3 (9)	5 (14)
Psychologist	2 (6)	2 (6)
Speech pathologist	1 (3)	2 (6)
Diabetes educator	1 (3)	1 (3)
Physiotherapist	1 (3)	1 (3)
Allied health assistant	3 (9)	0 (0)
Experience of dietitians employed (*n* = 34) or contracted (*n* = 33)		
Less than 2 years	20 (59)	10 (30)
2 to <5 years	19 (56)	14 (42)
5 to <10 years	16 (47)	11 (33)
10 years or more	13 (38)	12 (36)

Remuneration for dietitians employed or contracted by private practices is provided in Tables S1 and S2, respectively. Practices that contracted dietitians commonly remunerated dietitians by commission only (*n* = 21; 64%), with commission paid to dietitians most frequently 50% of client fees (*n* = 8; 27%), followed by 60% (*n* = 6; 20%), 65% (*n* = 3; 35%), and 70% (*n* = 3; 10%) of client fees. Facilities or support most frequently provided by the practice to contracted dietitians included appointment management (e.g., booking and reminders; *n* = 30; 91%), patient resources (*n* = 26; 79%), and clinical space (e.g., facility or room; *n* = 24; 73%). Informal mentoring (*n* = 22; 67%) was frequently provided, while clinical supervision (*n* = 14; 42%) and formal mentoring (*n* = 7; 21%) were less frequently provided. For the purposes of the survey, formal mentoring specifically referred to mentoring within the APD Program,^30^ while clinical supervision was defined as a structured, professional relationship that allows the supervisee to reflect on their practice, explore ethical issues, and develop critical thinking skills.^31^ Several practices (*n* = 4) also provided marketing, referral generation, and social media support.

Frequency of business and dietitian performance evaluation is presented in Table 3. Forty percent of practices (*n* = 61) periodically evaluated business performance—weekly, monthly, quarterly, six‐monthly, or annually—through a structured process. This evaluation must have examined outcomes, such as financial measures, client retention, waitlist length, or number of consultations, over a defined period. Practices that evaluated client satisfaction and/or experience via a survey (*n* = 45) did so after consultations (*n* = 16; 36%), ad hoc (*n* = 13; 29%), annually (*n* = 6; 13%), 6‐monthly (*n* = 5; 11%), monthly (*n* = 2; 4%) or upon completion of service (*n* = 2; 4%). One practice had a kiosk in the reception area where clients could provide feedback at any time. No practice evaluated client satisfaction via formal interview or focus group. Approximately one‐quarter of practices periodically evaluated general (*n* = 44; 29%) or clinical (*n* = 36; 23%) performance of dietitians. General performance was defined as how well an individual fulfilled their responsibilities based on their position within the practice, while clinical performance specifically referred to measurable changes in client outcomes (e.g., HbA1C, gastrointestinal symptoms) resulting from dietetic care. Notably, self‐reflection or evaluation of outcomes for individual clients during a consultation did not qualify as clinical performance. Significant positive correlations were observed between gross revenue and frequency of business (*τ*_b_ = 0.231, *p* < 0.001) and general dietitian (*τ*_b_ = 0.338, *p* < 0.001) performance evaluation, but not with dietitian clinical performance evaluation (*τ*_b_ = 0.120, *p* = 0.078). Small associations were observed between business structure and frequency of business (*χ* ^2^ (4) = 19.64, *p* < 0.001) and dietitian general (*χ* ^2^ (4) = 15.763, *p* = 0.003) performance evaluation, but not with clinical (*χ* ^2^ (4) = 7.44, *p* > 0.05) performance evaluation. The assumption of expected values was not met for clinical performance evaluation.

**TABLE 3:** Frequency of business and dietitian performance evaluation conducted by private dietetics practices (*n* = 154).

Characteristic	*n* (%)
Evaluation of business performance	
Never	48 (31)
Ad hoc	45 (29)
Weekly	5 (3)
Monthly	27 (18)
Quarterly	14 (9)
6 monthly	5 (3)
Annually	10 (6)
Method of client satisfaction evaluation	
Informally	133 (86)
Electronic survey	41 (41)
Paper‐based survey	5 (3)
Telephone survey	4 (3)
Other	6 (4)
Evaluation of dietitian general performance	
Never	62 (40)
Ad hoc	45 (29)
Weekly	5 (3)
Monthly	11 (7)
Quarterly	9 (6)
6 monthly	10 (6)
Annually	9 (6)
Other	3 (2)
Evaluation of dietitian clinical performance	
Never	71 (46)
Ad hoc	47 (31)
Weekly	6 (4)
Monthly	10 (6)
Quarterly	11 (7)
6 monthly	3 (2)
Annually	6 (4)
Frequency of dietitian professional development	
Never	13 (8)
Ad hoc	37 (24)
Weekly	21 (14)
Monthly	56 (36)
Quarterly	17 (11)
6 monthly	3 (2)
Annually	4 (3)
Other	3 (2)

Products or services offered by practices are presented in Table S3 and included client consultations (*n* = 146; 95%), followed by client programs (*n* = 34; 22%), menu reviews and/or development (*n* = 26; 17%), books or resources (*n* = 23; 15%), programs and support for other dietitians (*n* = 21; 14%), nutritional supplements (*n* = 16; 10%), programs and support for non‐dietetic professionals (*n* = 14; 9%), meal replacement products (*n* = 6; 4%), subscription to app or website (*n* = 5; 3%), portion control tools (*n* = 5; 3%), and exercise (*n* = 1; 1%) or blood glucose or pressure (*n* = 1; <1%) equipment. Participants (*n* = 14; 9%) also reported other services used to generate revenue, including consulting, presentations, workshops, content creation, and monetised social media content. Most participants (*n* = 90; 58%) offered more than one product or service.

Consultation characteristics are presented in Table 4. Most practices offered online (*n* = 121; 82%) client consultations, while one practice delivered consultations in a small rural hospital. Approximately one quarter of practices delivered consultations in general medical (*n* = 43; 29%), dietetics (*n* = 39; 27%), or multidisciplinary clinics (*n* = 37; 25%). The median number of practices serviced for each practice type was: three general medical practices (IQR 3.5), one multidisciplinary practice (IQR 1.0), and one specialist practice (IQR 1.0). A small proportion of practices delivered services to aged care facilities (*n* = 19; 13%), servicing a median of three facilities (IQR 6.0). Challenges described in short‐answer responses by participants who delivered consultations related to communication and coordination with healthcare providers and others involved in client care (*n* = 10), namely in regard to access to health and medical information and implementation of the care plan.

**TABLE 4:** Characteristics of consultations for private dietetics practices that generated revenue via this service (*n* = 147).

Characteristic	
Clients instructed to complete pre‐assessment^a^, *n* (%)	
No	62 (43)
Yes, sometimes	27 (19)
Yes, always	55 (38)
Type of pre‐assessment, *n* (%)	
Health and medical information	73 (89)
Goals and expectations	49 (60)
Food‐related information	41 (50)
Symptom information	23 (28)
Validated screening tool	10 (12)
Mental health or mood information	5 (6)
Other	3 (4)
Consultation setting, *n* (%)	
Online	121 (82)
Mobile and/or home	60 (41)
General medical clinics	43 (29)
Dietetics clinics	39 (27)
Multidisciplinary clinics	37 (25)
Specialist medical clinics	21 (14)
Aged care facilities	19 (13)
Gym or fitness centres	6 (4)
Other	1 (<1)
Consultation duration (initial)^b^, *n* (%)	
30 min	6 (4)
40 min	3 (2)
45 min	20 (14)
50 min	10 (7)
55 min	2 (1)
60 min	83 (59)
70 min	1 (<1)
75 min	3 (2)
90 min	11 (8)
Consultation duration (review)^b^, *n* (%)	
15 min	1 (<1)
20 min	5 (4)
25 min	2 (1)
30 min	80 (57)
35 min	1 (<1)
40 min	3 (2)
45 min	19 (14)
50 min	10 (7)
60 min	18 (13)
Consultation funding, *n* (%)	
Client	142 (97)
Medicare	133 (90)
Private health insurance	118 (80)
NDIS	115 (78)
DVA	69 (47)
Organisation	36 (24)
Compensation fund or scheme	35 (23)
Other	8 (5)
Fee based on dietitian experience^c^, *n* (%)	
No	24 (42)
Yes	21 (37)
Not applicable (i.e., dietitians similarly experienced)	12 (21)
Fees for initial consultations, mean (95% CI)	
Standard	$173 (±$9)
Standard (adjusted to rate per hour)	$188 (±$9)
Extended	$212 (±$22)
Least experienced dietitian	$188 (±$24)
Most experience dietitian	$244 (±$33)
Fees for standard initial consultations, mean (95% CI)	
Online only	$212 (±$36)
New South Wales	$174 (±$15)
Queensland	$158 (±$18)
Victoria	$168 (±$17)
Western Australia	$163 (±$31)
Australian Capital Territory	$193 (±$63)
South Australia	$156 (±$68)
Tasmania^d^	–
Fees for review consultations, mean (95% CI)	
Standard	$113 (±$7)
Standard (adjusted to rate per hour)	$185 (±$8)
Extended	$160 (±$13)
Least experienced dietitian	$111 (±$19)
Most experience dietitian	$130 (±$28)
Fees for standard review consultations, mean (95% CI)	
Online only	$133 (±$24)
New South Wales	$107 (±$12)
Queensland	$102 (±$11)
Victoria	$124 (±$16)
Western Australia	$104 (±$22)
Australian Capital Territory	$95 (±$87)
South Australia	$94 (±$36)
Tasmania^d^	–

^a^ *n* = 144 (data missing for *n* = 4 participants).

^b^ *n* = 139 being practices that did not alter consultation length.

^c^ *n* = 57 being practices that employed or contracted one or more dietitians.

^d^ Results for Tasmania not reported to protect participant anonymity due to *n* = 1.

Initial consultations were most frequently 60 min (*n* = 83; 59%) or 45 min (*n* = 20; 14%), as presented in Table 4, while review consultations were most frequently 30 min (*n* = 80; 57%) or 45 min (*n* = 19; 14%). Six practices altered the consultation duration based on dietitian experience. For these practices, consultations for the least experienced dietitians ranged from 60 to 75 min for initial and 30 to 60 min for review consultations, and consultations for the most experienced dietitians ranged from 20 to 60 min for initial and 20 to 45 min for review consultations. Many practices offered extended consultations (i.e., longer than standard consultations) for initial and review (*n* = 48; 33%) consultations, or initial (*n* = 8; 6%) or review (*n* = 24; 16%) consultations only.

The mean fee for a standard initial consultation was AU$173 (±$9 95% CI) and ranged from $90 to $380. The average fee for a standard review consultation was AU$113 (±$7 95% CI) and ranged from $90 to $180. For those practices that offered extended consultations, the average fee for an extended initial consultation was AU$212 (±$22 95% CI) and ranged from $105 to $490, while the average fee for an extended review consultation was AU$160 (±$13 95% CI) and ranged from $80 to $280. Several practices billed via an hourly rate for extended consultations that was applied pro‐rata or in 15‐min increments. Some practices (*n* = 21) varied the consultation fee based on dietitian experience. For the least experienced dietitians, the standard initial consultation fee was AU$188 (±$24 95% CI; range $105–260) and the standard review consultation fee was AU$111 (±$19 95% CI; range $70–190). For the most experienced dietitians, the standard initial consultation fee was AU$244 (±$33 95% CI; range $159–340) and the standard review consultation fee was AU$130 (±$28 95% CI; range $70–295). Some participants (*n* = 6) described the challenge of developing a fee schedule that appropriately compensates for the time taken to deliver the service, balancing financial viability with affordability for the client.

Most practices that offered consultations accepted clients via Medicare (*n* = 133; 90%) or National Disability Insurance Scheme (*n* = 115; 78%) referral, with fewer accepting Department of Veterans Affairs clients (*n* = 69; 47%). Of those practices that accepted National Disability Insurance Scheme clients, only 18 (16%) were registered providers. Most practices (*n* = 101; 76%) did not adjust their consultation fee for Medicare‐subsidised clients (i.e., scheduled fee accepted as full or part payment), as presented in Table 5. Only a small proportion of practices that accepted Medicare‐subsidised clients charged the scheduled fee (i.e., bulk billed) for initial (*n* = 16; 12%) and review (*n* = 18; 14%) consultations, as illustrated in Table 5. The average fee for initial and review consultations for Medicare‐subsidised clients, combining both practices that adjusted and did not adjust their fees, was AU$163 (±$11 95% CI) and AU$105 (±$7 95% CI), respectively. Some participants (*n* = 6) described experiencing pressure from referrers and clients to bulk bill Medicare‐subsidised services. Most practices (*n* = 118; 89%) did not shorten consultations for Medicare‐subsidised clients, with a small number shortening initial (*n* = 1; <1%), review (*n* = 4; 3%), or initial and review (*n* = 7; 5%) consultations for these clients. Others (*n* = 3; 2%) only shortened consultations for bulk billed clients.

**TABLE 5:** Characteristics for private dietetics practices that accepted Medicare‐subsidised clients for individual (*n* = 133) and/or group (*n* = 3) services.

Characteristic	
Consultation fee adjusted for individual services (*n* = 133), *n* (%)	
No	101 (76)
Yes, for both initial and review consultations	25 (19)
Yes, for initial consultations only	5 (4)
Yes, for review consultations only	2 (2)
Item numbers used for individual services (*n* = 133), *n* (%)	
Chronic disease, dietitian, in person (10954)	117 (88)
Chronic disease, telehealth or phone (93000, 93013)	107 (80)
Eating disorders, dietitian, in person (82350)	74 (56)
Eating disorders, dietitian, telehealth or phone (93074, 93108)	57 (43)
Chronic disease Aboriginal or Torres Strait Islander, dietitian, in‐person (81320)	21 (16)
Chronic disease, case conference services (10955, 10957, and 10959)	13 (10)
Chronic disease Aboriginal or Torres Strait Islander, telehealth or phone (93048, 93061)	6 (5)
Eating disorders, case conference services (80176, 80177, 80178))	2 (2)
Fees for initial consultations, mean (95% CI)	
Standard	$163 (±$11)
Fees for review consultations, mean (95% CI)	
Standard	$105 (±$7)
Item numbers used for group services for type‐2 diabetes (*n* = 3), *n* (%)	
Initial assessment, dietitian, in person (81120)	3 (100)
Group services, dietitian, in person (81125)	3 (100)
Initial assessment, dietitian, telehealth or phone (93284, 93286)	2 (66)
Group services, dietitian, telehealth or phone (93285)	2 (66)

Most practices that offered client programs had one (*n* = 9; 24%) or two (*n* = 14; 41%) programs, though one business had over 20 unique programs. Client programs were most frequently developed by the practice (*n* = 30; 83%), while a smaller number were developed by another organisation (*n* = 4; 11%) or were delivered as Medicare‐subsidised group services for people with type‐2 diabetes (*n* = 3; 8%). Client programs developed by practices most frequently included educational content (*n* = 44; 86%) or consultations (*n* = 33; 65%), with others including email (*n* = 29; 57%) or telephone support (*n* = 13; 26%), meal plans (*n* = 21; 41%), support groups (*n* = 13; 26%), dietary analysis (*n* = 12; 24%), or a formal class (*n* = 7; 14%). Client programs developed by the practice were most frequently delivered online or via telehealth (*n* = 21; 70%), dietetic clinics (*n* = 7; 23%), other organisations (*n* = 5; 17%), or childcare facilities (*n* = 2; 7%). At least one practice delivered programs in each of the following areas: general, specialist, or multidisciplinary clinics, schools, or gym or fitness centres. No practices delivered programs in aged care facilities. Fees for client programs varied considerably, ranging from AU$25–150 per participant to AU$500–$3000 per program, though most were funded by the client (*n* = 25; 83%), an organisation (*n* = 12; 40%) or private health insurance (*n* = 8; 27%).

## 4 DISCUSSION

This study described private dietetics practices operating in Australia using data captured in the inaugural survey for the national dataset for Australian private practice dietetics. Private practices were primarily sole traders that delivered client consultations, earning less than AU$149 999 in annual revenue. Business and dietitian performance evaluation did not frequently occur, though evaluation frequency was positively associated with business revenue and structure (i.e., sole trader, partnership, company or trust). Most practices accepted clients via publicly funded schemes; some practices adjusted prices for these clients, while a small number bulk billed their service. This study offers the first insight into private practice dietetics in Australia through a business lens, complementing past work done at an individual level.^27^

The greatest challenge identified by participants related to ensuring the practice generated adequate and consistent revenue, a challenge similarly experienced by physiotherapists.^32^ While sustainability of business revenue is a complex issue,^33^, ^34^, ^35^ establishing effective and robust referral streams is critically important given that many participants engaged in Medicare‐subsidised programs.^36^, ^37^ These programs rely on general practitioners to initiate care via referral, which was identified as a barrier both by participants in the current study and previous research.^15^, ^17^ Research has found that general practitioners are more likely to refer a client to a dietitian if they have participated in nutrition education;^38^ however, nutrition education within medical curricula is limited.^39^, ^40^ Enhancing nutrition education in medical curricula and continuing professional development may improve referrals to dietitians, in addition to enhancing care coordination and communication, which was similarly raised as a challenge by participants in the current study.

Most practices in the current study did not evaluate business or dietitian performance periodically, despite evaluation being positively associated with revenue. Previous work has highlighted barriers to the collection of data to support business and clinical evaluation, including time and the lack of suitable and efficient systems.^19^ The National Safety and Quality Primary and Community Healthcare Standards,^41^ which informed a model of quality management for dietitians,^18^ highlight the importance of business systems to support evaluation in the context of safety and quality. However, dietitians have raised concerns about balancing paid and unpaid work in their practice and highlighted the need for support to develop business competencies.^25^ This is particularly pertinent given the recommendation from the Scope of Practice Review, which explored the barriers and enablers primary care professionals face in performing their full scope of practice, that practice accreditation be implemented.^5^ Participation in practice accreditation would potentially place further strain on private practices, highlighting the need for support (e.g., education, mentoring) to implement effective and efficient business systems to improve quality while minimising investment of unpaid time.

Eating disorders was the specialisation most frequently reported by practices in the current study, a finding that stands in contrast to the last national workforce study, conducted in 2013, where eating disorders was not listed as a common condition treated.^27^ This finding is understandable with the observed growth in Medicare claims for dietetic eating disorder services since they were introduced in 2019,^37^ evidence that private practice dietitians respond to changes in the regulatory environment. Given that most businesses are sole traders that do not employ or contract other dietitians, credentialling, such as that offered by the Australia and New Zealand Academy for Eating Disorders,^42^ and supervision for dietitians who work with this vulnerable population is important. Similarly, regulatory change in aged care may also impact private practice dietetics, with the strengthened Aged Care Standards, which come into effect in July 2025, requiring a menu review at least annually.^43^ The current study is the first to describe the proportion of private practices that deliver care in aged care settings, with 13% of practices delivering consultation services in this setting and 17% identifying menu reviews as a service; by comparison, residential aged care facilities (RACF) were not identified as a practice setting by dietitians in Ball et al.^27^ While dietitians perceive that being employed by an RACF improves integration,^44^ private practices still play a significant role in servicing this market segment, with many dietitians in a recent survey contracted to aged care facilities either independently or through a private practice.^45^ Similar to eating disorders, however, dietitians must be supported to develop knowledge and skills to deliver effective and safe care.^45^

This study has several strengths, namely the representative sample of practices that participated in terms of geographic distribution and gender, making this the most comprehensive description of Australian private practice dietetics currently available. Sample representativeness suggests that results may be generalisable. However, despite attempts to reach all private dietetics practices in Australia, some practices were inevitably missed. Consequently, future iterations of the survey will aim to be inclusive of practices that were not included in the initial register of practices or who commence operating in the future. We encourage owners of private practices to contact the authors to ensure they receive information about future data collection. Better understanding of private practices via the national dataset will not only identify areas for practice improvement for business owners but also will provide much needed data for the profession to advocate for change in terms of education and resources.

This study provides contemporary insights into the business context of Australian private practice dietetics. With a significant proportion of the dietetics workforce working in this setting, it is essential that dietitians operating private practices are supported to develop business acumen, both pre‐ and post‐dietetic credentialling. In turn, dietitians will strengthen their ability to implement sound business systems that support sustainable practice, enabling more efficient care delivery and coordination, better balancing of clinical and business activities, and future‐proofing the private practice workforce.

## AUTHOR CONTRIBUTIONS

All authors contributed to the conceptualization and design of the study. AK collected data. All authors contributed to the analysis and interpretation of results and to writing the manuscript. All authors critically reviewed the manuscript and approved the final version submitted for publication.

## FUNDING INFORMATION

Professor Ball and Dr. Kirkegaard were supported by an NHMRC Investigator Grant. All researchers were supported by the UQ Centre for Community Health and Wellbeing, which is part‐funded by Springfield City Group.

## CONFLICT OF INTEREST STATEMENT

Dr Amy Kirkegaard is an Associate Editor of *Nutrition & Dietetics*. She was excluded from the peer‐review process and all decision making regarding this article. The manuscript was managed throughout the review process by the Journal's Editor‐in‐Chief. All other authors have no conflicts of interest to declare.

## ETHICS STATEMENT

This study was approved by The University of Queensland Human Research Ethics Committee (Ref. No.: 2023/HE001399).

## Supporting information


**TABLE S1:**Remuneration for dietitians employed in private dietetics practices (*n* = 34).

**TABLE S2:**Remuneration and support for dietitians contracted by private dietetics practices (*n* = 33).

**TABLE S3:**Products or services offered by for private dietetics practices (*n* = 147).

## Data Availability

The data that support the findings of this study are available from the corresponding author upon reasonable request.
