# Supplementary material for: The business of dietetics: Results from the national Australian private practice dietetics dataset
Source: Nutr Diet. 2025 Aug 7;83(2):200–10. doi: 10.1111/1747-0080.70036 (PMC13096668; doi:10.1111/1747-0080.70036)
Supplement: Supplementary file 1 — TABLE S1:Remuneration for dietitians employed in private dietetics practices (n = 34). [file NDI-83-200-s001.docx]

**Table S1.** Remuneration for dietitians employed in private dietetics practices (n=34)

|  | **<2 years** | **2 to <5 years** | **5 to <10 years** | **>10 years** |
| --- | --- | --- | --- | --- |
| Annual salary (excluding superannuation and bonuses) |  |  |  |  |
| Less than $50,000 | 1 (5) |  |  |  |
| $50,000–59,999 | 4 (20) |  |  |  |
| $60,000–69,999 | 7 (35) |  | 1 (6) |  |
| $70,000–79,999 | 7 (35) | 9 (47) | 3 (19) |  |
| $80,000–89,999 | 1 (5) | 8 (42) | 6 (38) |  |
| $90,000–99,999 |  | 2 (11) | 4 (25) | 5 (38) |
| $100,000–109,999 |  |  | 2 (13) | 4 (31) |
| $120,000–129,999 |  |  |  | 3 (23) |
| $190,000–199,999 |  |  |  | 1 (8) |
