# Supplementary material for: The business of dietetics: Results from the national Australian private practice dietetics dataset
Source: Nutr Diet. 2025 Aug 7;83(2):200–10. doi: 10.1111/1747-0080.70036 (PMC13096668; doi:10.1111/1747-0080.70036)
Supplement: Supplementary file 2 — TABLE S2:Remuneration and support for dietitians contracted by private dietetics practices (n = 33). [file NDI-83-200-s003.docx]

**Table S2.** Remuneration and support for dietitians contracted by private dietetics practices (n=33)

|  | **n (%)** |
| --- | --- |
| Dietitian remuneration |  |
| Commission only | 21 (64) |
| Commission and time-based rate | 8 (24) |
| Time-based rate only | 3 (10) |
| Other | 1 (3) |
| Commission ratio (dietitian:practice) |  |
| 100:0 | 1 (3) |
| 87:13 | 1 (3) |
| 70:30 | 3 (10) |
| 65:35 | 3 (10) |
| 60:40 | 6 (20) |
| 55:45 | 2 (7) |
| 50:50 | 8 (27) |
| 47:53 | 1 (3) |
| 45:55 | 2 (7) |
| 42.5:57.5 | 1 (3) |
| 40:60 | 1 (3) |
| 30:70 | 1 (3) |
| Facilities or support provided |  |
| Appointment management | 30 (91) |
| Patient resources | 26 (79) |
| Clinical space | 24 (73) |
| Informal mentoring | 22 (67) |
| Computer equipment or software | 21 (64) |
| Professional development | 19 (58) |
| Clinical supervision | 14 (42) |
| Formal mentoring | 7 (21) |
| Other | 4 (12) |
