# Supplementary material for: The business of dietetics: Results from the national Australian private practice dietetics dataset
Source: Nutr Diet. 2025 Aug 7;83(2):200–10. doi: 10.1111/1747-0080.70036 (PMC13096668; doi:10.1111/1747-0080.70036)
Supplement: Supplementary file 3 — TABLE S3:Products or services offered by for private dietetics practices (n = 147). [file NDI-83-200-s002.docx]

**Table S3.** Products or services offered by for private dietetics practices (n=147)

| **Characteristic** | **n (%)** |
| --- | --- |
| Product or service offered |  |
| Client consultations | 146 (95) |
| Client programs | 34 (22) |
| Menu reviews and/or development | 26 (17) |
| Books or resources | 23 (15) |
| Professional programs and support for dietitians | 21 (14) |
| Nutritional supplements | 16 (10) |
| Professional programs and support for non-dietetics professionals | 14 (9) |
| Meal replacement products | 6 (4) |
| Subscription to app or website | 5 (3) |
| Portion control tools | 5 (3) |
| Exercise equipment | 2 (1) |
| Blood glucose or pressure monitors | 1 (<1) |
| Other | 14 (9) |
